# Solar Trees: First Large‐Scale Demonstration of Fully Solution Coated, Semitransparent, Flexible Organic Photovoltaic Modules

**DOI:** 10.1002/advs.201500342

**Published:** 2015-12-14

**Authors:** Stephane Berny, Nicolas Blouin, Andreas Distler, Hans‐Joachim Egelhaaf, Michal Krompiec, Andreas Lohr, Owen R. Lozman, Graham E. Morse, Lana Nanson, Agnieszka Pron, Tobias Sauermann, Nico Seidler, Steve Tierney, Priti Tiwana, Michael Wagner, Henry Wilson

**Affiliations:** ^1^ Merck Chemicals Ltd.Chilworth Technical CentreUniversity ParkwaySO16 7QDSouthamptonUK; ^2^ Merck Chemicals KGaAFrankfurter Strasse 25064293DarmstadtGermany; ^3^ BELECTRIC OPV GmbHLandgrabenstr. 9490443NürnbergGermany

**Keywords:** interface, organic photovoltaic modules, polymer, roll to roll, stability

## Abstract

**The technology behind a large area array of flexible solar cells** with a unique design and semitransparent blue appearance is presented. These modules are implemented in a solar tree installation at the German pavilion in the EXPO2015 in Milan/IT. The modules show power conversion efficiencies of 4.5% and are produced exclusively using standard printing techniques for large‐scale production.

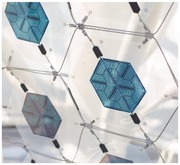

In this Communication, we present the technology behind the solar tree installation at the German pavilion, Milan EXPO, which represents a paradigm shift in solar technology, as it incorporates a large area array of flexible solar cells with a unique design and semitransparent blue appearance. The modules show power conversion efficiencies of 4.5% and were produced exclusively using common printing techniques.

High performance organic photovoltaic (OPV) polymers have been recently reported in the literature, highlighting the potential of carbon‐based semiconductors as a realistic source of power generation. OPV modules have the potential for extremely fast payback times for both energy, carbon, and cost versus other solar technologies.^1^, ^2^, ^3^ In terms of absolute device performance, 3rd Gen PV technologies are lagging behind approaches based on Si, CIGS, etc., but offer inherent advantages in flexibility, form factors, colour, transparency, lightweight, indoor efficiency, low‐light efficiency, and off‐axis performance. In the case of building integrated PV (BIPV), the inherent freedom of design and versatile adaptability allows OPV to better serve functional and aesthetic demands from architects as compared to classic PV technologies. Examples are the unique choice of colours, shapes, and degrees of transparency, which strongly improves the prospects to comply with preferred façade designs, and the straightforward compatibility with upcoming membrane architecture due to its flexible nature.^4^ Approaching the production of modules via solution‐based polymers also opens up cost‐effective and scalable roll‐to‐roll (R2R) manufacturing techniques with low environmental impact.^5^, ^6^

The target of Merck Chemicals has been to develop high performance OPV donor polymers. By carefully tailoring the chemical and physical properties of the materials, the requirements of commercially viable, thin film, flexible solar modules can be met, allowing integration in a sustainable way (**Figure** **1**). One of the products of this research program is the new series of blue donor polymers based around PBTZT‐stat‐BDTT‐8 from the Lisicon series, which offers:

- High solubility and uniform coatings from a range of solvents (including nonhalogenated). The formulations can be used with multiple lab‐scale coating techniques such as spin‐coating, doctor blade coating, bar‐coating, or high‐throughput production techniques such as slot‐die coating.
- High OPV device performance in different device architectures (regular and inverted, with a range of interlayers) and over a wide range of active layer thicknesses. This enables a broad processing window for robust and reproducible solution‐based R2R production processes.
- Potential for cost effective commercial‐scale production and integration into modules.

**Figure 1:**
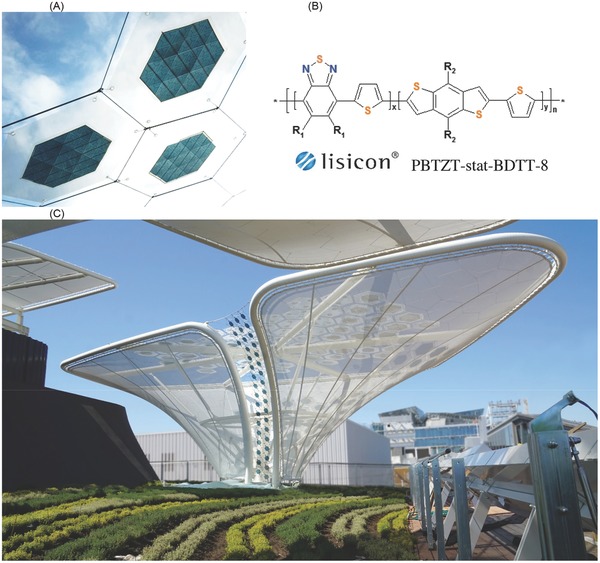
Images of A) a commercial module fabricated by BELECTRIC OPV, B) chemical structure of the polymer PBTZT‐stat‐BDTT, and C) large scale deployment of BELECTRIC OPV modules at the Universal Exhibition Milan 2015.^7^, ^8^

PBTZT‐stat‐BDTT‐8 addresses many of the current technical challenges toward the commercialization of OPV that have been recently outlined^9^, ^10^ and offers a real alternative to P3HT. From a materials standpoint, PBTZT‐stat‐BDTT‐8 can offer up to 9% PCE on the cell level, establishing it as a top tier OPV polymer along with the PTB7 family,^11^, ^12^, ^13^ the PBDTBDD and PDBT‐T1 family,^14^, ^15^ the PPDT2FBT family,^16^ the PffBT4T‐2OD family,^17^ and recently DT‐PDPP2T‐TT.^18^ Most importantly, state‐of‐the‐art progress from lab cell to fully R2R modules producing 4.5% power conversion efficiency (PCE) from semitransparent modules is demonstrated herein. This pioneering work has already shown the highest reported performance of flexible R2R modules of fully solution‐processed single junction OPV design^19^, ^20^ leading to its first demonstration of flexible, semitransparent OPV modules integrated into the Universal EXPO in Milan, 2015.^7^, ^8^, ^21^

PBTZT‐stat‐BDTT‐8 is the outcome of polymer backbone optimization from our previously reported polymer system based on the PBTZTBDTT family, and is composed of substituted benzodithiophene, thiophene, and benzothiadiazole (Figure 1b).^22^ The “R_1_” and “R_2_” were selected from a library of polymers and simultaneously enhances solubility, power conversion efficiency,^23^ and device stability.^24^, ^25^ The result is a royal‐blue polymer with a band gap of 1.7 eV and absorption peaks at 600 nm and 640 nm in the solid state. The frontier molecular energy levels of PBTZT‐stat‐BDTT‐8 were measured by cyclic voltammetry at −3.7 and −5.4 eV. The molecular weight of PBTZT‐stat‐BDTT‐8 is used to alter formulation viscosity to match the technique requirements of a particular printing or coating method. In the present case of doctor blade and slot‐die coating, the molecular distribution was fixed at *M*_n_ = 22.2 kg mol^−1^ (*M*_w_ = 49.5 kg mol^−1^, *Đ*_M_ = 2.23).^26^ PBTZT‐stat‐BDTT‐8 is widely soluble in common organic solvents which are used for bulk heterojunction (BHJ) processing, including 1,2‐dichlorobenzene, chlorobenzene, chloroform, *o*‐xylene, 1‐methylnaphthalene, tetraline, and mesitylene, amongst others.^27^, ^28^ High PCE can be obtained by optimizing formulations in any of these solvents although we have determined that mixtures of *o‐*xylene and tetraline provide a good balance of PCE, solubility, fullerene solubility, viscosity and coating uniformity, while being entirely halogen free.^28^, ^29^

As clearly illustrated by Krebs and Jorgensen, a true demonstration of a commercial high performance OPV polymer can only be performed on large areas, by many laboratories, and using commercially relevant printing techniques.^10^ Our evaluation and optimization commenced on small area cells measuring 0.04 cm^2^. To facilitate the transfer from a lab scale process to a R2R process, ink formulation and device optimizations were performed using a doctor blade coating technique rather than spin‐coating. Here we employed solution processable PEDOT:PSS as a hole‐transporting layer (HTL, Clevios P VP AL 4083), PV‐E001 as an electron‐transporting layer (ETL, Merck), and 1,2‐dichlorobenzene as a screening solvent for the standard architecture. Combining PBTZT‐stat‐BDTT‐8 with PC_60_BM (Lisicon PV‐A600) in 1,2‐dichlorobenzene with a solids loading of 30 mg mL^−1^ (1:2 weight ratio of PBTZT‐stat‐BDTT‐8:PV‐A600) allowed the fabrication of state‐of‐the‐art regular stack OPV devices giving 9.3% PCE peak (8.5% PCE average, **Figure** **2**). This is one of the few commercially available polymers which is currently able to produce >9% PCE using an industrially relevant coating technique, off the shelf solution processed interlayers, and PC_60_BM as an acceptor material. The EQE of these devices exceed 70% with a peak of 80% at 668 nm in the standard architecture. Exceptionally high FF are produced in the range of 74%–70% even at active layer thicknesses of 500 nm (**Figure** **3**b). These performances can be consistently reproduced in our laboratory.

**Figure 2:**
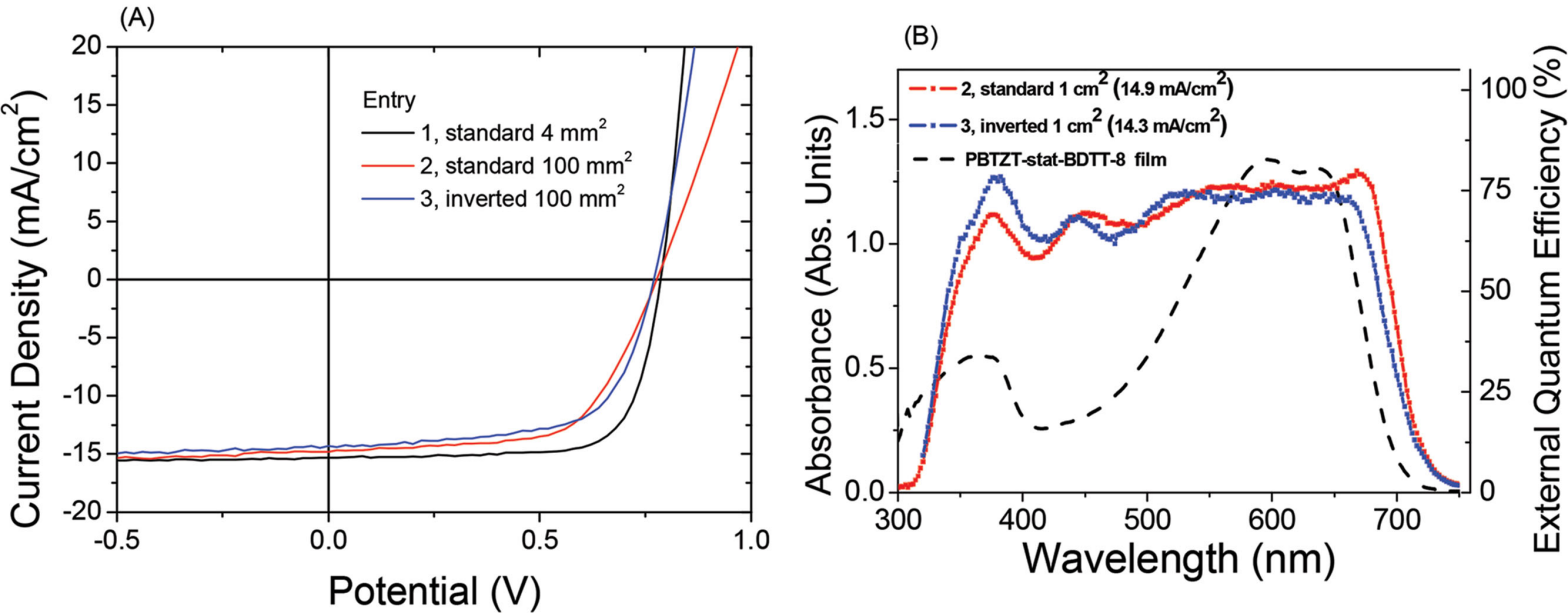
A) Current density as a function of applied potential for devices of type 1–3. B) External quantum efficiency for devices of type 2 (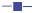) and 3 (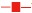) as well as an overlaid absorption spectra for PBTZT‐stat‐BDTT‐8 in the solid state.

**Figure 3:**
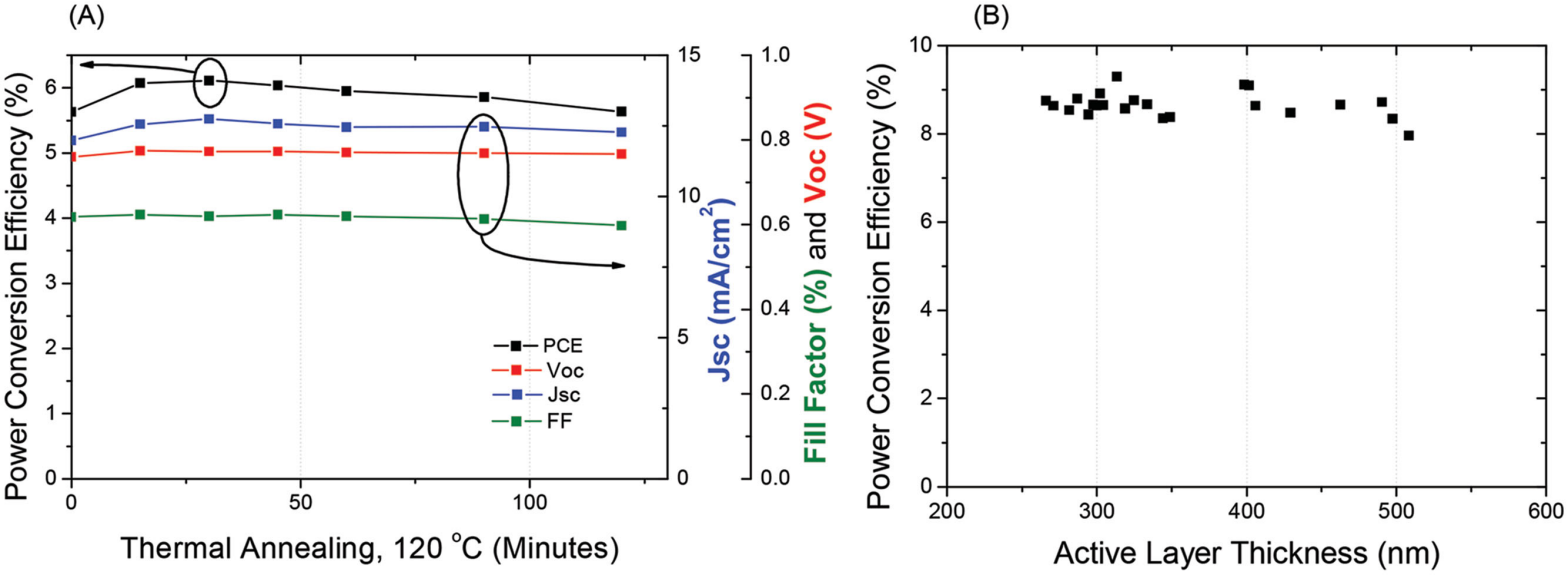
A) Thermal stability measured at 120 °C for devices of type 4 over 2 h showing the changes in power conversion efficiency (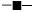), short circuit current (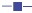), fill factor (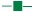), and open circuit voltage (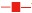). B) Effect of thickness on power conversion efficiency for devices of type 1 illustrating a wide processes window.

The next stage in development was to transition to systems that would highlight any challenges of Lisicon PBTZT‐stat‐BDTT‐8 integration into commercially viable mass‐produced OPV prototypes. It was therefore essential not only to measure the effects of moving to larger active areas and to plastic substrates in view of R2R OPV processes, but also to move from dichlorobenzene to nonhalogenated solvents for the active formulation in order to satisfy the requirements for industrial mass‐production. In this case, work was carried out on both device architectures, the inverted stack being fabricated by using PV‐E002 as the ETL in combination with PEDOT:PSS (HTL Solar Clevios 388). We combined PBTZT‐stat‐BDTT‐8 and PV‐A600 (30 mg mL^−1^ and 1:1.5 weight ratio) in a mixture of tetralin (12.5 vol%) and *o*‐xylene (87.5 vol%); the solvent mixture giving a suitable balance of solubility, performance, and viscosity.^29^, ^30^

Moving to large area devices produces additional losses from an increase in the series resistance across the cell, leading to a lowering of the performance.^9^, ^31^, ^32^, ^33^ Devices at 1.00 cm^2^ give peak PCEs at 7.5% (7.2% average PCE) for the inverted stack architecture. The main losses from the large area devices arise from a reduction in fill factor stemming from an increase in series resistance, for which the bottom cell design and conductivity are key contributors as highlighted by the difference between the regular and inverted stack behaviors at forward bias (Figure 2A). Inverted stack devices at 0.27 cm^2^ and prototyped on ITO‐metal‐ITO (IMI) coated PET exhibit a decrease in the overall performance driven by the *J*_sc_ compared to the equivalent device on glass (**Table** **1**). PBTZT‐stat‐BDTT‐8 allows thick active layers to be coated, therefore compensating for most of the optical losses of the substrates, while maintaining high FF and PCE (Figure 3B).

**Table 1:** Device performance of the different device and module architectures. Average performances of at least ten pixels are mentioned in between brackets, champion performances without

Type	Architecture (integration)	Substrate	TCO	HTL	ETL	Electrode [μm]	Active area [cm^2^]	*V*_oc_ [V]	*J*_sc_ [mA cm^−2^]	FF [%]	*PCE* [%]
1	Standard (cell)	Glass	ITO 100 [nm]	4083	PV‐E001	Ag+Al 0.1+0.1 Evaporated	0.04	0.78 [0.77]	16.3 [15.2]	74 [73]	9.3 [8.5]
2	Standard (cell)	Glass	ITO 100 [nm]	4083	PV‐E001	Ag 0.1 Evaporated	1.00	0.78 [0.77]	14.9 [14.0]	63 [61]	7.3 [6.6]
3	Inverted (cell)	Glass	ITO 100 [nm]	388	PV‐E002	Ag 0.1 Evaporated	1.00	0.80 [0.79]	14.3 [13.5]	68 [65]	7.5 [7.2]
4	Inverted (cell)	PET	IMI	388	PV‐E002	Ag 0.1 Evaporated	0.27	0.77 [0.76]	12.6 [12.1]	67 [63]	6.5 [6.0]
5	Inverted (module cell)	PET	IMI	388 + pH1000	PV‐E002	Ag 0.1 Evaporated	0.27	0.78 [0.77]	9.8 [9.6]	64 [62]	4.8 [4.6]
6	Inverted (trigon module‐9 cells)	PET	IMI	388 + pH1000	PV‐E002	Ag Screen‐printing	114.5	7.0 [6.8]	119.7a) [117.2a)]	62 [61]	4.5 [4.3]

^a)^ *I*_sc_ values are given for the modules type 6.

The final stage of integration of PBTZT‐stat‐BDTT‐8 into modules was performed using a R2R process. The substrates are made of prestructured IMI on PET, and the HTL and ETL were PEDOT:PSS HTL Solar Clevios 388 and PV‐E002, respectively. The HTL was customized in order to ensure printability on top of the BHJ and its compatibility with an additional transparent conductive layer (TCL) of PEDOT:PSS PH1000, helping current extraction when used in conjunction with a Ag grid electrode. The performances were first checked on cells measuring 0.27 cm^2^, which were cut out of the roll of OPV modules printed by R2R and completed using evaporated Ag. In this configuration, performances of 4.8% PCE can be reached from the opaque cells. This validates the particular architecture of the OPV stack and its processing by R2R, even if some losses in performance originate from the lower *J*_sc_ values. Those losses are attributed to the high thickness of the bilayer of HTL, which reduce the contribution of the evaporated Ag electrode to the *J*_sc_ by means of multireflection processes, and which can also change the light distribution in the BHJ. Moving from an evaporated electrode to an interdigitated Ag structure, the latter being needed to attain a semitransparent final product, leads to performances as high as 4.5% PCE prior to encapsulation. Remarkably, this BELECTRIC OPV process and design minimizes losses of the *V*_oc_ and FF versus lab‐scale devices, despite the stack being fully printed.^34^, ^35^, ^36^ In real world tests, these bifacial semitransparent modules are expected to exhibit a slight increase in efficiency by harvesting light from both sides.^37^ The modules consist of a combination of 54 cells in total, grouped as 9 serially connected cells, which are then connected in parallel (**Figure** **4**). A record 4.3% average PCE for the module was achieved for this semitransparent system. The use of high precision laser structuring allows the increase of the geometrical fill factor (GFF) to a high level of 95%.^34^, ^35^, ^36^ This contributes not only to improving the module performance, but also significantly improves the aesthetic appeal of the final modules, which is key to delivering building integrated photovoltaics (BIPV, Figure 1).^38^ More than 250 m^2^ of modules, with an average performance of 4% PCE and an average transparency of ≈20% were fabricated for installation in the solar trees at the German pavilion in the 2015 Universal Expo in Milan.

**Figure 4:**
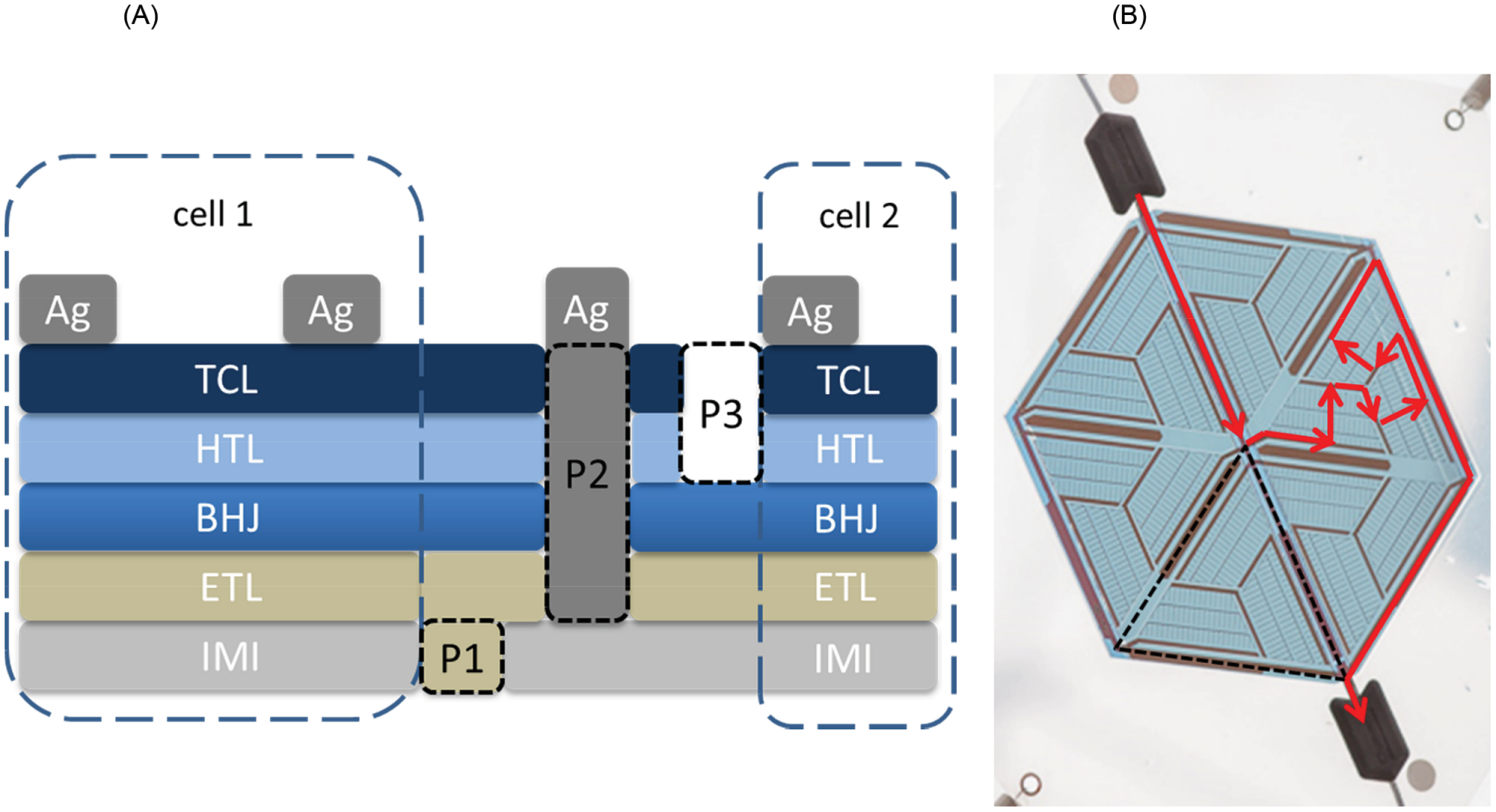
A) Schematic of the modules layout. The interconnect P2 of two sub‐cells is made by laser scribing. The distance of two cells (beginning of P1 to end of P3) is 1 mm. B) Close‐up of a small Trigon module installed in an EXPO tree. One of the small modules consists of six submodules, each in a triangular shape (dashed line), which are all connected in parallel. One triangular module is made of nine subcells, all connected in series. The red arrow symbolizes the current pathway for one distinct triangular submodule from the anode to the cathode.

Besides performance, excellent thermal stability of the OPV modules is a prerequisite for their integration into many end products. For instance OPV elements embedded in glass (e.g., façade elements) require stability for 2 h at 120 °C in the lamination process.^38^ Thus these conditions serve as good test standard and were carried out on inverted single cells on PET (same configuration as Type 4, Figure 3). The device was periodically measured during this test in order to track any changes in performance during thermal stress. After an initial 15 minutes of heating, an increase in *J*_sc_ is observed causing an increase in PCE while FF and *V*_oc_ remain unchanged. Beyond the 60 minutes timeframe, a slow decrease in FF and *J*_sc_ result in a minor loss in PCE, reverting back to its starting performance of 5.6%. Overall excellent thermal stability is observed for PBTZT‐stat‐BDTT‐8 in this device configuration showing no significant change over 2 h of heating, thereby allowing for its integration within laminated supports. This result is attributed to the intrinsic thermal stability of PBTZT‐stat‐BDTT‐8, and to its incorporation into a finely tuned stack and solvent mixture.

To validate the usage of the trigon modules under real conditions during the Universal EXPO in Milan 2015, light‐soaking experiments of the systems were run under 1 Sun illumination without showing any significant decrease in PCE over the first 100 h. More data will be collected during the Universal EXPO and will be reported at a later stage. In the meantime, further engineering in terms of donor and acceptor materials and formulations are ongoing in order to enable any OPV market segments and printing processes with PBTZT‐stat‐BDTT‐8.

In conclusion, PBTZT‐stat‐BDTT‐8 demonstrates a combination of features that are essential for the integration into sustainable and adaptable commercial OPV products, such as membrane architecture applications or as laminates in glass‐glass elements.

On the lab‐scale, this material offers efficiencies higher than 9% PCE, which is comparable to the best polymers reported to date. In addition to high performance, excellent processability is also critical when moving to fully solution‐based, R2R fabrication of OPV modules: PBTZT‐stat‐BDTT‐8 is able to reach high performance with commercially viable acceptor materials, such as PC_60_BM and using a variety of coating technologies, broad thickness range, different cell sizes, and with formulations in a variety of nonhalogenated solvents. This unique combination of high optoelectronic performance and wide processing window enabled the fabrication of >250 m^2^ flexible “trigon” modules reaching an average performance of 4.3% PCE, while maintaining a high level of transparency and aesthetic appeal. In real world tests, these bifacial modules are expected to show an increase in efficiency due to light harvesting from both sides.^37^ Furthermore, initial tests on the thermal stability of these OPV modules meet requirements for lamination protocols for glass‐based BIPV products, thereby accessing the important market of façade applications (in the context of the upcoming nearly‐zero energy building directive 2010/31/EU). The large deployment of this material during the Universal EXPO 2015 in Milan is the first example of large‐scale production of customized high performing solution‐processable OPV systems, a clear demonstration that OPV is a viable technology to complement and contribute to the energy mix in a decarbonized global economy.

## Experimental Section

*Materials*: PBTZT‐stat‐BDTT‐8, PV‐A600, PV‐E001, and PV‐E002 were purchased from Merck. ITO‐patterned substrates were purchased from Zencatec. PET‐based flexible substrates comprising a transparent conductive layer, namely an ITO‐metal‐ITO (IMI) sandwich structure, were purchased from Materion and used for R2R module fabrication. Clevios HTL Solar 388, Clevios P VP Al 4083, Clevios PH 1000, and the Ag ink used for screen‐printing were purchased from Heraeus. The solvents as well as the metals to evaporate were bought through Sigma‐Aldrich.

*Polymer Characterization*: Gel permeation chromatography (GPC) was performed at an elution rate of 1 mL min^−1^ with 1,2,4‐trichlorobenzene (Aldrich) at 160 °C through a PSS polefin 10 μm (50 × 8 mm) precolumn and three PSS polefin 10 μm (300 × 8 mm) GPC columns. The polymers were analyzed with a refractive index detector calibrated with narrow polystyrene standards. Samples were prepared at a concentration of 3 mg mL^−1^. Cyclic voltammograms were recorded using a Princeton Applied Research VersaSTAT 4 potentiostat. Films of the polymers were cast from a concentrated chloroform solution onto a platinum wire working electrode. Voltammograms were recorded in an anhydrous acetonitrile solution containing 0.1 M NBu_4_^+^ BF_4_^−^ electrolyte with a platinum wire counter electrode and 0.1 m Ag AgNO_3_^−1^ in acetonitrile reference electrode. The solutions were purged with N_2_ gas and referenced to an external ferrocene solution which was also used to calculate the ionization potential (IP) and electron affinity (EA) positions (*E*_orbital_ = −(*E*_onset_ + 5.1) eV).^39^

*OPV Cells and Modules Fabrication*: All the devices at lab‐scale were fabricated by doctor blade coating under ambient conditions. The only exception was the HTL in the regular stack, which was coated by spin‐coating, under ambient conditions. Prepatterned substrates of IMI or ITO were cleaned with a sequence of successive baths in acetone, IPA, and distilled water. Substrates were then dried with compressed air.

In the case of the small cell sizes in the regular stack architecture (0.04 cm^2^, type 1), the HTL made of Clevios P VP Al 4083 was filtered (PVDF coated, 0.45 μm pore size and 25 mm diameter, from Merck Millipore) and then added to distilled water in a 1:1 ratio per volume. The formulation was then coated by spin‐coating at 4000 rpm for 60 s to reach a thickness of 20 nm, and annealed at 120 °C for 30 minutes in air. The active layer formulation was made by mixing PBTZT‐stat‐BDTT‐8 with Merck PV‐A600 in 1,2‐dichlorobenzene with a solids loading of 30 mg mL^−1^ (1:2 weight ratio of PBTZT‐stat‐BDTT‐8:PV‐A600). The formulation was heated for 2 to 3 h on a hotplate at 90 °C under stirring, after which it was ready to be coated on top of the HTL. The coating parameters for the active layer formulations are: speed = 30 mm s^−1^, temperature = 70 °C, gap = 100 μm, volume = 70 μL. The film was dry after 2 minutes at 70 °C. The solution of PV‐E001 was maintained at room temperature and a volume of 30 μL was coated on top of the BHJ by doctor blade at room temperature, with a speed of speed = 20 mm s^−1^, gap = 50 μm, after which a successive layer of Ag (100 nm) and Al (100 nm) were evaporated at a standard rate of 1–5 Å s^−1^. In the case of large cell size (100 mm^2^, type 2), a different solvent system was used composed of 87.5% *o*‐xylene and 12.5% tetralin with a solids loading of 30 mg mL^−1^ (1:1.5 weight ratio of PBTZT‐stat‐BDTT‐8:PV‐A600).

For inverted stack architecture devices (types 3 and 4), 140 μL of PV‐E002 was coated by doctor blade from a formulation at room temperature at a speed of 5 mm s^−1^, a doctor blade temperature of 80 °C, and a gap = 575 μm, on top of the substrates. The BHJ was processed from a nonhalogenated solvent mixture as described previously and the HTL Solar 388 was coated on top by doctor blade from a formulation maintained at room temperature, with a speed of 30 mm s^−1^, a gap of 575 μm, and the doctor blade being at a temperature of 65 °C. An evaporation of Ag (100 nm) was then used to complete the stack.

All modules were fabricated on a R2R machine as previously described.^40^ The machine comprises different un‐winders, edge guides, backing rolls, IR heaters, impingement dryers, three slot‐die heads, and one rotary screen printing unit. A camera system and a UV/VIS spectrometer were installed for quality analysis of the laser lines and coated layers. The IMI substrates, rolls of 165 mm × 150 m, were P1 structured with a Rofin 1064 nm Nd:YVO_4_ laser. PV‐E002 was stirred for 30 minutes at room temperature, the active materials in a solvent mixture of 87.5% Xylene and 12.5% Tetralin with a solid content of 37.0 mg mL^−1^ (PBTZT‐stat‐BDTT‐8:PV‐A600 ratio of 1:1.5) was stirred overnight at 90 °C, and HTL Solar 388 and PH 1000 were stirred at room temperature for 2 h. PV‐E002 was slot‐die coated with a resulting dry layer thickness of 5 nm. The BHJ was coated with a heated syringe, tube, and coating head with a resulting dry layer thickness of 250 nm. Both layers were coated in one run with 2 m min^−1^ and additional drying steps were applied. HTL Solar 388 and PH 1000 were coated with a dry layer thickness of 70 nm and 150 nm, respectively, in one coating run at 1 m min^−1^. Afterward, P2 and P3 was laser structured with the above‐mentioned laser and the semitransparent top electrode was screen printed from Ag ink with a dry layer of 6 μm. The final modules were roll‐to‐batch encapsulated between two barrier foils with the use of an epoxy glue.

*OPV Device and Module Characterization*: The areas of the solar cells were defined by the area of the top electrode evaporated on top of the device. They were checked by optical microscope to avoid shadow effects. The *J*_sc_ values reported were extrapolated from EQE measurements (Newport). The solar simulator was a Newport 91160, giving AM1.5G irradiance and 1Sun intensity, as calibrated with a Si‐photodiode (Newport 91150V). In the case of plastic devices and modules, current–voltage characteristics of the solar cells and modules were recorded under a calibrated Oriel solar simulator with xenon lamp providing 1 sun (AM 1.5G, 1000 W m^−2^) by using a Keithley 2400 source measurement unit in combination with a Keithley 7001 Multiplexer system. External quantum efficiency was measured with a custom‐made setup. Absorption spectra were performed with an OceanOptics USB2000+. Thickness measurements were done by mechanical profilometry using a Dektak Alpha‐Step 500.
